# Assessment of Haemodynamic Response to Nonselective Beta-Blockers in Portal Hypertension by Phase-Contrast Magnetic Resonance Angiography

**DOI:** 10.1155/2017/9281450

**Published:** 2017-06-15

**Authors:** Natasha McDonald, David M. L. Lilburn, Neil J. Lachlan, Gillian Macnaught, Dilip Patel, Arjun N. A. Jayaswal, Peter C. Hayes, Scott I. Semple, Jonathan A. Fallowfield

**Affiliations:** ^1^MRC Centre for Inflammation Research, Queen's Medical Research Institute, University of Edinburgh, 47 Little France Crescent, Edinburgh EH16 4TJ, UK; ^2^BHF Centre for Cardiovascular Science, Queen's Medical Research Institute, University of Edinburgh, 47 Little France Crescent, Edinburgh EH16 4TJ, UK; ^3^Glasgow Royal Infirmary, 84 Castle Street, Glasgow G4 0SF, UK; ^4^Department of Radiology, Royal Infirmary of Edinburgh, 51 Little France Crescent, Edinburgh EH16 4SA, UK; ^5^University of Oxford Centre for Clinical Magnetic Resonance Research (OCMR), Level 0, John Radcliffe Hospital, Oxford OX3 9DU, UK; ^6^Liver Unit, Royal Infirmary of Edinburgh, 51 Little France Crescent, Edinburgh EH16 4SA, UK

## Abstract

A significant unmet need exists for accurate, reproducible, noninvasive diagnostic tools to assess and monitor portal hypertension (PHT). We report the first use of quantitative MRI markers for the haemodynamic assessment of nonselective beta-blockers (NSBB) in PHT. In a randomized parallel feasibility study in 22 adult patients with PHT and a clinical indication for NSBB, we acquired haemodynamic data at baseline and after 4 weeks of NSBB (propranolol or carvedilol) using phase-contrast MR angiography (PC-MRA) in selected intra-abdominal vessels. T1 mapping of liver and spleen was undertaken to assess changes in tissue composition. Target NSBB dose was achieved in 82%. There was a substantial reduction from baseline in mean average flow in the superior abdominal aorta after 4 weeks of NSBB therapy (4.49 ± 0.98 versus 3.82 ± 0.86 L/min, *P* = 0.03) but there were no statistically significant differences in flow in any other vessels, even in patients with >25% decrease in heart rate (47% of patients). Mean percentage change in liver and spleen T1 following NSBB was small and highly variable. In conclusion, PC-MRA was able to detect reduction in cardiac output by NSBB but did not detect significant changes in visceral blood flow or T1. This trial was registered with the ISRCTN registry (ISRCTN98001632).

## 1. Introduction

Structural changes within the cirrhotic liver, combined with portal and systemic haemodynamic changes, lead to portal hypertension (PHT) which underlies the majority of clinical manifestations and complications of cirrhosis such as varices, ascites, hepatorenal syndrome, and encephalopathy. Currently, hepatic venous pressure gradient (HVPG) measurement is the only validated method of assessing PHT and evaluating the effect of pharmacological interventions. The prognostic value of PHT measurement at different stages in the natural history of cirrhosis is well established, with cut-off values for the development of complications (HVPG > 10 mmHg) and variceal rupture (HVPG > 12 mmHg) [1, 2]. However, HVPG measurement is invasive and expensive and is neither accessible nor used widely outside of specialist centres. A recommendation from the Baveno V Consensus Workshop on Methodology of Diagnosis and Therapy in PHT was to identify noninvasive tools for measuring PHT, which could have clinical utility for monitoring changes in PHT over time or in response to treatment [3].

A number of approaches have been investigated for the noninvasive assessment of PHT encompassing routine laboratory tests (e.g., platelets to spleen ratio [4]), serum markers of inflammation and fibrosis (e.g., sCD163 and Enhanced Liver Fibrosis test [5]), quantitative assays of liver function (e.g., dual cholate clearance [6], indocyanine green retention [7]), and imaging techniques (e.g., transient elastography [8]) which have all shown varying levels of diagnostic accuracy. Recently, magnetic resonance imaging (MRI) based techniques have shown promise for investigating differential visceral blood flow in the hyperdynamic circulation of patients with cirrhosis [9] and in quantifying PHT. For example, MRI derived hepatic blood flow parameters and azygous flow have been shown to correlate with portal pressure [10] and variceal size [11], respectively. Furthermore, a recent study suggested a predictive model of HVPG based on the combination of MRI acquired splenic artery velocity and liver T1 relaxation time [12]. These MRI measurements can be performed on 1.5-tesla or 3-tesla scanners, potentially without contrast agents, no breath-hold scans, and short scan times, all features which increase the potential for the widespread adoption of the technique across healthcare systems.

Nonselective beta-blockers (NSBB) reduce HVPG and are, therefore, an established treatment in both primary and secondary prophylaxis of variceal bleeding in cirrhosis, either in combination with endoscopic band ligation or as an alternative [13]. It has been repeatedly shown that less than 50% of patients achieve a successful haemodynamic response to propranolol, which is in turn associated with an increased risk of variceal bleeding [14, 15]. Perhaps unsurprisingly, HVPG-guided drug therapy in PHT was recently shown to achieve greater reduction in portal pressure leading to better patient outcomes, including survival [16]. In addition to its well-documented effect on portal pressure, data from animal models also suggests that carvedilol may have an antifibrotic and anti-inflammatory effect in liver parenchyma [17, 18], potentially modifying extracellular matrix composition and influencing liver T1 relaxation time.

We hypothesised that quantitative MRI derived haemodynamic and structural markers, acquired noninvasively in a single scan session, could be used to inform which patients respond to NSSB therapy. Here, we report the findings of an initial small feasibility study.

## 2. Patients and Methods

### 2.1. Study Design and Patient Population

This was a single-centre, open-label, parallel study conducted at the Royal Infirmary of Edinburgh from January 2015 to March 2016. The study was conducted in accordance with the ethical principles of the Declaration of Helsinki 2013 and Good Clinical Practice guidelines. It was approved by NHS Lothian Research and Development and the South East Scotland Research Ethics Committee 02 (REC Reference: 14/SS/1050). All patients gave written informed consent.

22 patients requiring prophylaxis of variceal haemorrhage for cirrhotic PHT were randomized 1 : 1, by sealed opaque envelope, to either carvedilol or propranolol treatment. Study inclusion criteria were age 18–80 and presence of liver cirrhosis and PHT where commencement of NSBB was clinically indicated. Study exclusion criteria were contraindication to NSBB therapy (such as moderate to severe asthma) or MRI scan; contraindication to administration of gadolinium-based MRI contrast (including eGFR < 30 mL/min); concomitant use of drugs used to treat PHT; previous TIPSS insertion; portal vein thrombosis; hepatocellular carcinoma; pregnancy or breastfeeding; and inability to obtain informed consent.

### 2.2. Assessments

Upon enrolment, information on liver disease aetiology, past medical history, medication and alcohol history, and results of the most recent upper gastrointestinal endoscopy were recorded. Patients underwent a physical examination and routine laboratory investigations (full blood count, coagulation screen, and liver and renal function tests), followed by the baseline MRI scan. Liver disease severity was also assessed at baseline according to the Model for End Stage Liver Disease (MELD) score and Child-Pugh score. Starting doses of once-daily NSBB were 6.25 mg for carvedilol and 80 mg for modified release propranolol. Patients' compliance with medication and adverse events monitoring were assessed at an initial follow-up visit after 1 week of NSBB therapy. Provided that NSBB were tolerated clinically and haemodynamically (resting heart rate (HR) ≥ 50 beats per minute (b.p.m), systolic blood pressure (SBP) ≥ 95 mmHg), the dose was escalated to the target once-daily dose of 12.5 mg of carvedilol or 160 mg of propranolol. Further treatment compliance and adverse events monitoring were assessed by weekly telephone consultations. After 4 weeks, whilst on the NSBB, the second MRI was performed. An interval of 4 weeks was chosen as haemodynamic responses to NSBB after chronic use exceed the acute response rate [19]. Consistent with previous landmark NSBB trial in PHT, treatment was targeted at a resting HR reduction of more than 25% from baseline [20]; this was defined as a clinical haemodynamic response to NSBB (HR responders).

### 2.3. MRI

Patients were fasted for at least 4 hours prior to MRI scans to avoid postprandial changes in splanchnic blood flow. All patients had an estimated glomerular filtration rate of more than 60 mL/min and serum creatinine levels within the normal range. The radiology team (radiographers, radiologists, and MRI physicists) were blinded to the treatment allocation.

MR imaging was obtained at the Edinburgh Imaging Facility at Queen's Medical Research Institute using a 3-tesla Verio MRI system (Siemens Healthcare, GmbH, Erlangen, Germany) with a combination of spine matrix and body matrix coil elements. Firstly, we used electrocardiogram-gated gadolinium (Gd; Gadavist 0.1 mmol/kg) contrast-enhanced MRA sequences to visualise the vessels and rapidly identify the appropriate cross-section view of the vessel of interest. Phase-contrast MR was then planned on the appropriate view in order to determine flow rates within that vessel, as previously described [9]. Assessment of flow rates was performed in the following vessels: proper hepatic artery, portal vein, superior mesenteric artery, superior aorta (acquired 2 cm above the coeliac trunk), inferior aorta (acquired 2 cm above the iliac bifurcation), renal arteries, and azygous vein, as previously described [9]. 2-D PC blood flow was analysed by two independent observers experienced in PC-MRA, using Siemens Argus Flow software, as previously described [9].

In addition, we assessed liver and spleen T1 relaxation time mapping using an established protocol [21].

### 2.4. MRI T1 Analysis

Analysis of the pre-Gd T1 maps of liver and spleen was performed, with the aim of identifying a change in the T1 value between the pre- and posttreatment scans. The images were analysed by a single operator using Liver*MultiScan*™ software (Perspectum Diagnostics, Oxford, UK). T1 values representing the mode of a segmented liver/spleen slice histogram were used for analysis. In addition, 3 regions of interest (ROI) were positioned manually within the liver/spleen T1 maps and the mean values of 3 ROIs were used for further analysis.

### 2.5. Statistical Analysis

A sample size of 20 patients per group was calculated as sufficient to create a 90% confidence interval on the mean change from baseline that would exclude the zero if a 25% change was observed for that parameter, assuming the observed baseline mean and standard deviation for the study was similar to the historically observed standard deviation [9]. Following problems with patient recruitment and extended study staffing, the target sample size was reduced to 10 patients per group (i.e., total of 20 completed patients), supported by blinded interim analysis on 16 completed patient datasets which indicated that the overall analysis of the reduced patient dataset would still generate meaningful and valuable results.

Statistical analysis was performed using GraphPad Prism version 6.0 (GraphPad Software, USA). Variables were summarised as mean ± standard deviation (SD) if normally distributed and as median with interquartile range (IQR) if not. A comparison of numerical variables between the groups was performed using paired Student's* t*-test when the samples were normally distributed and the Mann–Whitney* U*-test when not. Chi-square test was used to compare categorical data. Association between two continuous variables was assessed using the Pearson and Spearman correlation coefficient, as appropriate. The variability of the liver and splenic T1 data and the interobserver variability were assessed using Bland-Altman plots. A *P* value of less than 0.05 (two-tailed) was considered statistically significant throughout.

## 3. Results

### 3.1. Demographic and Clinical Characteristics of Study Participants

22 patients were recruited during the study period (Table 1). Most were male (82%) with a mean ± SD age of 56 ± 9 years. Commonest aetiologies of liver disease were alcohol (36%) and nonalcoholic fatty liver disease (32%). The majority of patients (59%) were Child-Pugh class A, and median (IQR) MELD score was 10 (8–15). There were no differences in baseline characteristics between carvedilol and propranolol groups. 19 out of 22 participants had both baseline and repeat MRI scans (1 patient died from complications of cirrhosis during the study; 2 patients were unable to tolerate NSBB, both on propranolol). Target NSBB dose was achieved in 82% overall, but more frequently with carvedilol (100% carvedilol versus 64% propranolol; *P* = 0.03). A greater than 25% reduction in resting HR was achieved in 47% of participants who completed the study (56% in propranolol group and 40% in carvedilol group).

### 3.2. Quantification of the Regional and Visceral Blood Flow

**Figure 1:**
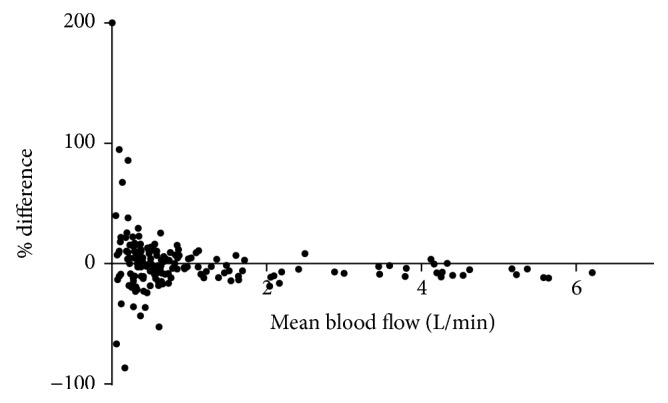
Bland-Altman plot of percentage differences in average blood flow measurements between the two observers against the mean blood flow. The bias between the 2 sets of measurements was small (0.2% or 0.05 L/min; limits of agreement −0.23 to 0.33).

There was excellent interobserver agreement for average blood flow assessment (Pearson correlation coefficient 1.0, *P* < 0.0001, and Bland-Altman analysis, Figure 1).

**Figure 2:**
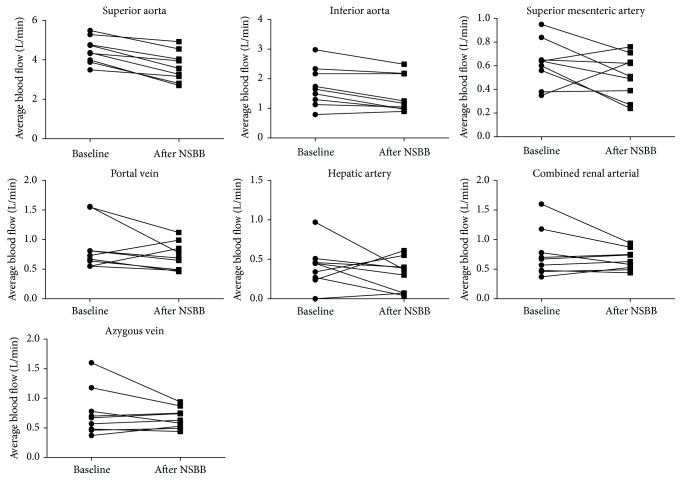
Individual PC-MRA derived blood flow measurements in heart rate responders at baseline and after 4 weeks of NSBB therapy (*n* = 9). There was a significant reduction in the blood flow in superior and inferior aorta after 4 weeks of NSBB therapy (*P* < 0.001 and 0.010, resp.). The changes in flow in all other vessels were not statistically significant. Data analysed by paired* t*-test.

**Figure 3:**
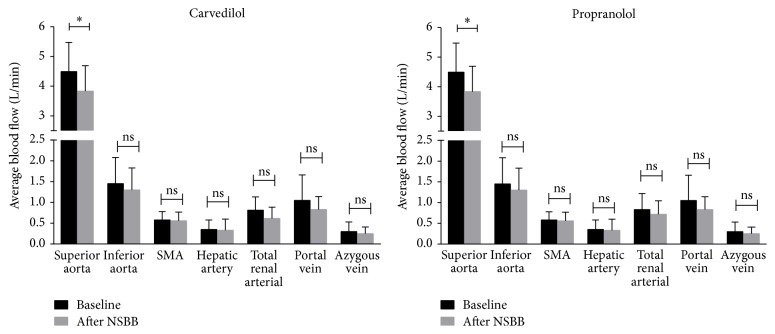
Baseline and 4-week post-NSBB PC-MRA derived blood flow measurements for individual vessels. Data represented as mean ± SD and analysed by paired* t*-test; ^*∗*^*P* < 0.05, ns: not significant. SMA: superior mesenteric artery.

There was a substantial reduction from baseline in mean average blood flow in the superior abdominal aorta after 4 weeks of NSBB therapy (4.49 ± 0.98 versus 3.83 ± 0.86 L/min, *P* = 0.029; *n* = 19; Table 2). In all other vessels, there was a downward trend in mean average flow after 4 weeks of NSBB therapy, although the reduction in flow was not statistically significant. In the HR responders, there was a significant reduction in mean average blood flow in the superior and inferior aorta (*n* = 9; Figure 2). There were no appreciable differences in blood flow changes between carvedilol and propranolol treated patients (Figure 3).

### 3.3. Liver and Spleen T1 Mapping

**Figure 4:**
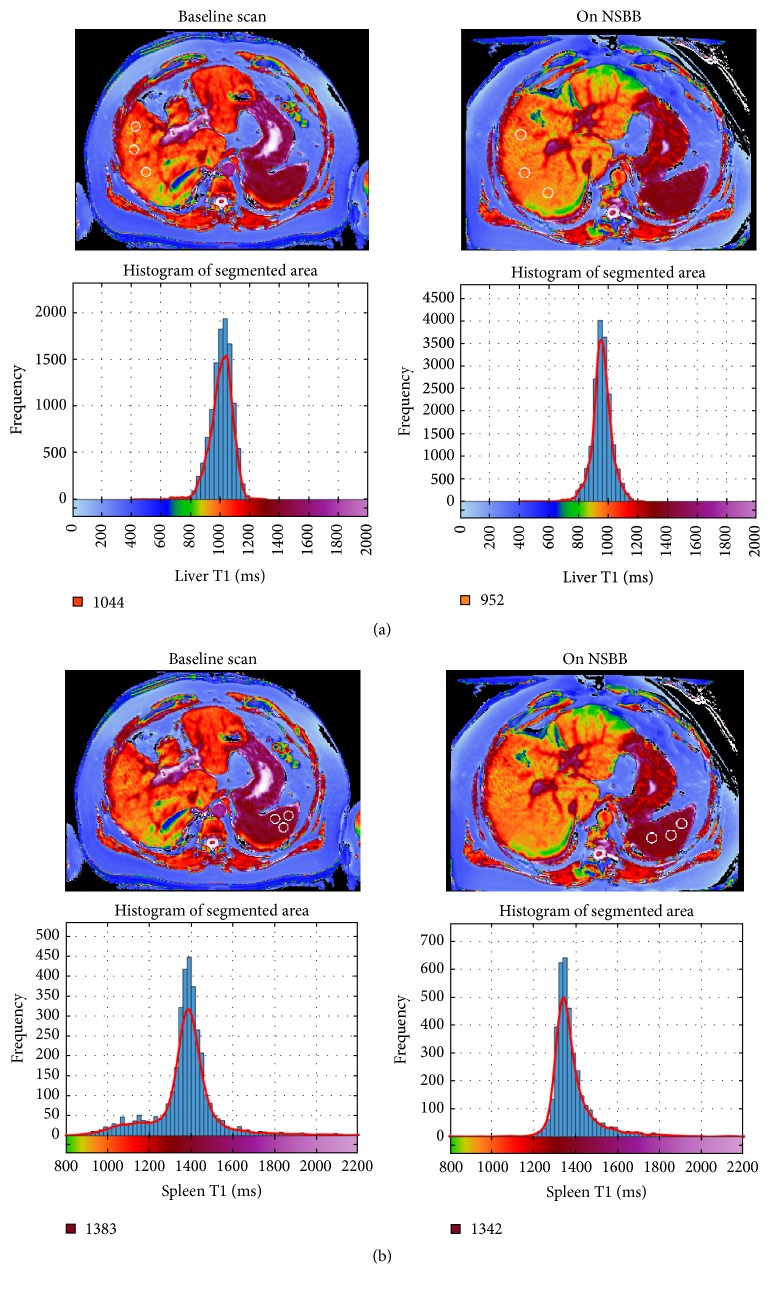
Axial T1 relaxation maps of liver (a) and spleen (b) before and after NSBB therapy. Top row shows representative image of liver (a) and spleen (b) T1 relaxation map from a single patient; position of the 3 ROIs is indicated by white circles. Bottom row shows corresponding segmented liver (a) and spleen (b) histograms and T1 colour scales from the same patient. Numbers below each histogram represent the mode T1.

**Figure 5:**
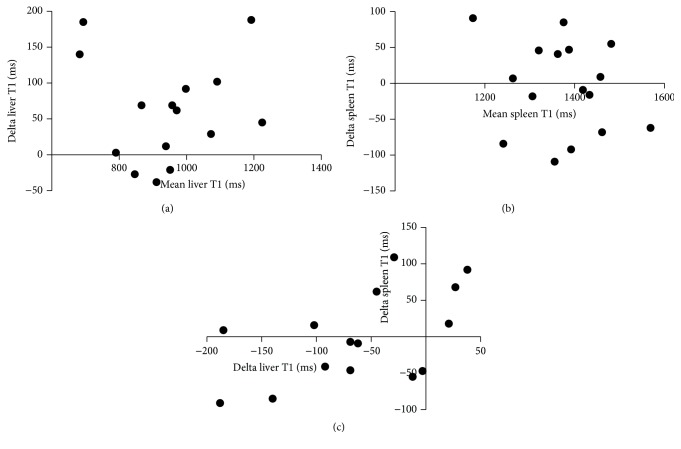
Absolute change in liver (a) and spleen (b) T1 (delta T1) after 4 weeks of NSBB therapy. Correlation of changes in delta T1 between liver and spleen (c). There was no consistent increase or decrease in liver or splenic T1 values as a result of NSBB treatment. In addition, there was no clear correlation found between changes in liver T1 and splenic T1. Bland-Altman analysis.

There was a strong agreement between the 2 methods (histogram and ROI) to estimate liver and spleen T1 values (Spearman's Rho = 0.98 for liver and 0.95 for spleen, *P* < 0.001 for both, *n* = 15). Representative screen shots of the axial T1 relaxation maps are shown in Figure 4. Overall, there was no consistent trend of change in liver or spleen T1 after 4 weeks of NSBB (Figure 5). There was no clear correlation between changes in liver T1 against spleen T1 (*n* = 15) (Figure 5(c)). The range of changes in T1 were similar in absolute values between the liver (1.2–193 ms) and spleen (18.5–99.2 ms). The mean ± SD percentage change in liver and spleen T1 following NSBB was small and highly variable (7.67 ± 6.8 for liver and 3.88 ± 2.6 for spleen).

## 4. Discussion

This is the first study to use PC-MRA as a noninvasive readout of response in an interventional study of the pharmacological treatment for PHT. Previous research has shown that this technique is reproducible and reliable [9, 11, 22, 23]. Importantly, all patients were able to tolerate the baseline and repeat scans, including patients with ascites. There were no reported adverse reactions to gadolinium contrast in this group of patients with advanced liver disease. We were able to obtain good quality flow measurements in all patients and all vessels as well as interpretable T1 relaxation maps of liver and spleen in 79% of patients who had both baseline and 4-week scans.

We confirmed previous reports that NSBB achieve the target HR response in around 50% of patients (47% overall in our study). The issue of compliance with medication was addressed by weekly monitoring (in person and via telephone) for the duration of the trial and supported by the fact that all 19 patients that completed the study achieved a significant reduction between baseline and 4-week HR (paired* t*-test *P* < 0.0001).

In this study, PC-MRA was able to detect a significant reduction in cardiac output by NSBB (as measured by superior aortic flow). This is a predictable effect of NSBB and, reassuringly, provides validation of the use of the PC-MRA methodology in this population. However, PC-MRA did not detect statistically significant changes in blood flow in other vessels, such as the expected reduction in portal inflow and splanchnic vasoconstriction. This may simply be a reflection of the small sample size for this study. Additionally, the HR responders showed a significant reduction in the average flow in the inferior aorta and a nonsignificant trend towards decrease in flow in all other vessels, again suggesting that a larger study may have shown larger and significant haemodynamic effects. Furthermore, it is also possible that the known variable clinical efficacy of NSBB contributed to the interpretation of results, especially since reduction in HR has previously not been found to correlate strongly with the HVPG response [24]. To establish, definitively, whether PC-MRA has sufficient sensitivity to monitor the response to NSBB, a larger study with concurrent HVPG measurements and/or clinical outcomes will be required.

The lack of a significant and consistent change in liver T1 values may be due to the relatively short interval between the 2 scans, thus not allowing sufficient time for remodelling of hepatic extracellular matrix that may occur as a result of proposed antifibrotic and/or anti-inflammatory effect of carvedilol. The absence of concurrent T2^*∗*^ mapping, which allows an iron correction to be applied to T1 values [21], may also be relevant, although significant intrasubject variation in tissue iron concentration during the 4-week follow-up period seems unlikely. In this study, T1 relaxation mapping was unable to detect reduction in liver and spleen perfusion, expected as a result of haemodynamic effects of NSBB. This could be due to either the lack of significant effect of NSBB on visceral blood flow or the dominant contribution of the liver/spleen fibrosis to the T1 readout and consequent lack of sensitivity of this technique to the changes in blood volume.

## 5. Conclusions

In this initial feasibility study, PC-MRA was able to detect a robust reduction in cardiac output by NSBB (as measured by superior aortic flow) but did not detect significant changes in visceral blood flow or T1 relaxation time in liver and spleen. A larger study, evaluating NSBB or TIPSS by PC-MRA and contemporaneous HVPG measurement, is now required to determine the true value of noninvasive MRI in this setting.

## Figures and Tables

**Table 1:** Baseline characteristics of the study population.

Patient characteristic	Treatment group			*P* value (propranolol versus carvedilol)
	Propranolol MR	Carvedilol	All patients	
Number	11	11	22	1.00
Age, years (mean ± SD)	56 ± 7	56 ± 10	56 ± 9	0.99
Male sex, *n* (%)	10 (91)	8 (73)	18 (82)	0.28
Aetiology of liver disease				
Alcoholic liver disease, *n* (%)	5 (45)	3 (27)	8 (36)	0.39
Nonalcoholic fatty liver disease, *n* (%)	3 (27)	4 (36)	7 (32)	0.66
Viral hepatitis, *n* (%)	0 (0)	2 (18)	2 (9.1)	0.15
Other, *n* (%)	3 (27)	2 (18)	5 (23)	0.62
Child-Pugh Score, *n* (%)				
Child-Pugh A	7 (64)	6 (55)	13 (59)	0.67
Child-Pugh B	2 (18)	3 (27)	5 (23)	0.62
Child-Pugh C	2 (18)	2 (18)	4 (18)	1.00
Baseline heart rate, bpm	78 (69–81)	82 (72–94)	79 (69–89)	0.14
Systolic BP, mm Hg	128 (116–146)	136 (115–150)	136 (115–150)	0.99
Splenomegaly, *n* (%)	8 (73)	8 (73)	16 (73)	1.00
Thrombocytopaenia, *n* (%)	9 (82)	10 (91)	19 (86)	0.55
Final beta blocker dose (mg)	160 in 7 patients 80 in 4 patients	12.5 in all	—	
Target beta blocker dose achieved, *n* (%)	7 (64)	11 (100)	18 (82)	**0.03**
Completed study, *n* (%)	9 (82)	10 (91)	19 (86)	0.55
Heart rate responders, *n* (%)	5^*∗*^ (56)	4 (40)	9 (47)	0.50

^*∗*^Heart rate response in propranolol group was observed in 5 patients; 3 on 80 mg of propranolol MR and 2 on 160 mg of propranolol MR.

**Table 2:** Baseline and 4-week post-NSBB blood flow. Data shown as mean ± SD and analysed by paired *t*-test (*n* = 19).

	Baseline blood flow (L/min)	4-week blood flow (L/min)	*P* value
Superior aorta	4.49 ± 0.98	3.83 ± 0.86	**0.029**
Inferior aorta	1.45 ± 0.63	1.30 ± 0.53	0.41
Superior mesenteric artery	0.58 ± 0.20	0.56 ± 0.21	0.76
Proper hepatic artery	0.35 ± 0.23	0.33 ± 0.27	0.85
Total renal arterial	0.82 ± 0.34	0.64 ± 0.32	0.079
Portal vein	1.05 ± 0.61	0.83 ± 0.31	0.17
Azygos vein	0.30 ± 0.23	0.25 ± 0.16	0.49
